# Correlation between serum phosphate and all-cause mortality in critically ill patients with coronary heart disease accompanied by chronic kidney disease: a retrospective study using the MIMIC-IV database

**DOI:** 10.3389/fcvm.2024.1371000

**Published:** 2024-05-31

**Authors:** Min He, Siyu Ren, Yongqi Lin, Xiaocong Zeng

**Affiliations:** ^1^ Department of Cardiology, The First Affiliated Hospital of Guangxi Medical University, Nanning, Guangxi, China; ^2^ Guangxi Key Laboratory Base of Precision Medicine in Cardiocerebrovascular Diseases Control and Prevention & Guangxi Clinical Research Center for Cardio-Cerebrovascular Diseases, Nanning, Guangxi, China; ^3^ School of Basic Medical Sciences, Guangxi Medical University, Nanning, Guangxi, China

**Keywords:** serum phosphate, coronary heart disease, chronic kidney disease, all-cause mortality, MIMIC-IV database

## Abstract

**Background:**

The adverse clinical endpoints of cardiovascular and kidney diseases are correlated with increased serum phosphate levels. However, in critically ill patients with coronary heart disease (CHD) accompanied by chronic kidney disease (CKD), the prognostic value of serum phosphate remains unclear.

**Methods:**

Patients' medical records from the Medical Information Mart for Intensive Care IV database who had concomitant CKD and CHD were classified into four distinct groups in this large retrospective observational cohort study based on the quartiles of serum phosphate levels. Vital status and the duration of hospital and ICU stays within the short-term follow-up periods of 30 and 90 days constituted the primary outcomes. All-cause mortality in the intensive care unit (ICU) and hospital constituted the secondary outcomes. Further, the Cox proportional hazard and restricted cubic spline (RCS) regression models were employed to ascertain how serum phosphate levels correlated with the primary outcomes. In addition, the occurrence rate of the secondary outcomes across the four quartiles was determined utilizing the Kaplan–Meier method.

**Results:**

Among the total 3,557 patients (67.6% male) included, the hospital and ICU all-cause mortality rates were 14.6% and 10%, separately. Higher quartiles of serum phosphate concentrations were associated with shorter short-term survival rates, as shown by the Kaplan–Meier curves. Additionally, the Cox proportional hazards analysis illustrated that serum phosphate was independently linked to a higher death risk in the hospital [HR, 1.10 (95% CI: 1.03–1.18), *P* = 0.007] and ICU [HR, 1.14 (95% CI: 1.07–1.22), *P* < 0.001]. Lastly, the RCS regression models suggested a robust non-linear correlation between serum phosphate concentrations and death risk in the ICU and hospital (both *P* for non-linearity <0.001).

**Conclusions:**

The prognostic value of serum phosphate is significant in critically ill patients with CHD accompanied by CKD. Furthermore, serum phosphate is potentially valuable for identifying patients with this concomitant condition.

## 1 Introduction

Coronary heart disease (CHD) is a major contributor to mortality globally (1). Although medical technology advancements have substantially improved the prognosis of cardiovascular diseases (CVDs) such as CHD in developed regions, CVD persists as the primary reason for admissions to the intensive care unit (ICU) and even death. Furthermore, there have been reports indicating that critically ill patients with CHD in the ICU are more prone to develop comorbidities and organ dysfunction (2). A recent clinical study highlighted the prevalence of CHD combined with chronic kidney disease (CKD) with a growing annual incidence rate (3). CKD is an irreversible condition characterized by progressive renal dysfunction and exhibits a strong correlation with cardiovascular risks in terms of pathology (4). Patients having CHD combined with CKD often experience relatively more severe cardiovascular symptoms and complications, such as heart failure (HF), acute myocardial infarction (AMI), and arrhythmias. Thus, the combined occurrence of these two diseases poses remarkable challenges to patient prognosis. Moreover, the intricate interplay and complexity of these two severe conditions may impact the efficiency and security of CHD treatments in the presence of CKD, necessitating cautious approaches in the clinical treatment of patients with both these diseases. Therefore, a critical need exists for studies exploring more convenient and effective predictors to identify patients at a higher risk for CHD with CKD at an early stage. These studies should primarily focus on enhancing patient prognosis and providing a sound scientific basis for making individualized treatment decisions.

Mineral metabolism is crucial in the physiological functioning of the renal and cardiovascular systems. In patients having CKD combined with CVD, abnormal mineral metabolism has been demonstrated to contribute to a higher death risk (5–7). Of these minerals, phosphate is an essential component in the normal physiological metabolism of humanity, serving a crucial function in the generation of energy, synthesis of DNA and RNA, and intracellular signal transduction. The precise regulation of serum phosphate entails complex interactions between various organs and tissues, among which the kidneys are critically involved (8). Higher serum phosphorus levels are linearly associated with an increased risk of developing renal failure (9). Numerous studies have identified abnormal phosphate homeostasis as a risk factor for CVD (10). Additionally, a retrospective investigation examining the link between CHD death risk and serum phosphate concentration revealed that increased baseline levels of serum phosphate were positively correlated with a higher death risk in populations with end-stage renal disease and impaired kidney function (11).

However, in critically ill patients with CHD accompanied by CKD, the prognostic value of serum phosphate levels is unclear. Therefore, the investigation objective was to retrospectively analyze the potential associations between serum phosphate and all-cause mortality in this specific patient population.

## 2 Manuscript formatting

### 2.1 Method

#### 2.1.1 Data sources

In this retrospective observational research, all included data were sourced from the Medical Information Mart for Intensive Care-IV (MIMIC-IV; version 2.2) database, which is a large, publicly open dataset administered and managed by the Laboratory for Computational Physiology of Massachusetts Institute of Technology. MIMIC-IV database contains the medical health records at the Beth Israel Deaconess Medical Center in the United States (12). After completing the online course and passing the exam, the author acquired access to the database (record ID: 57369428).

#### 2.1.2 Study population and data extraction

**Figure 1:**
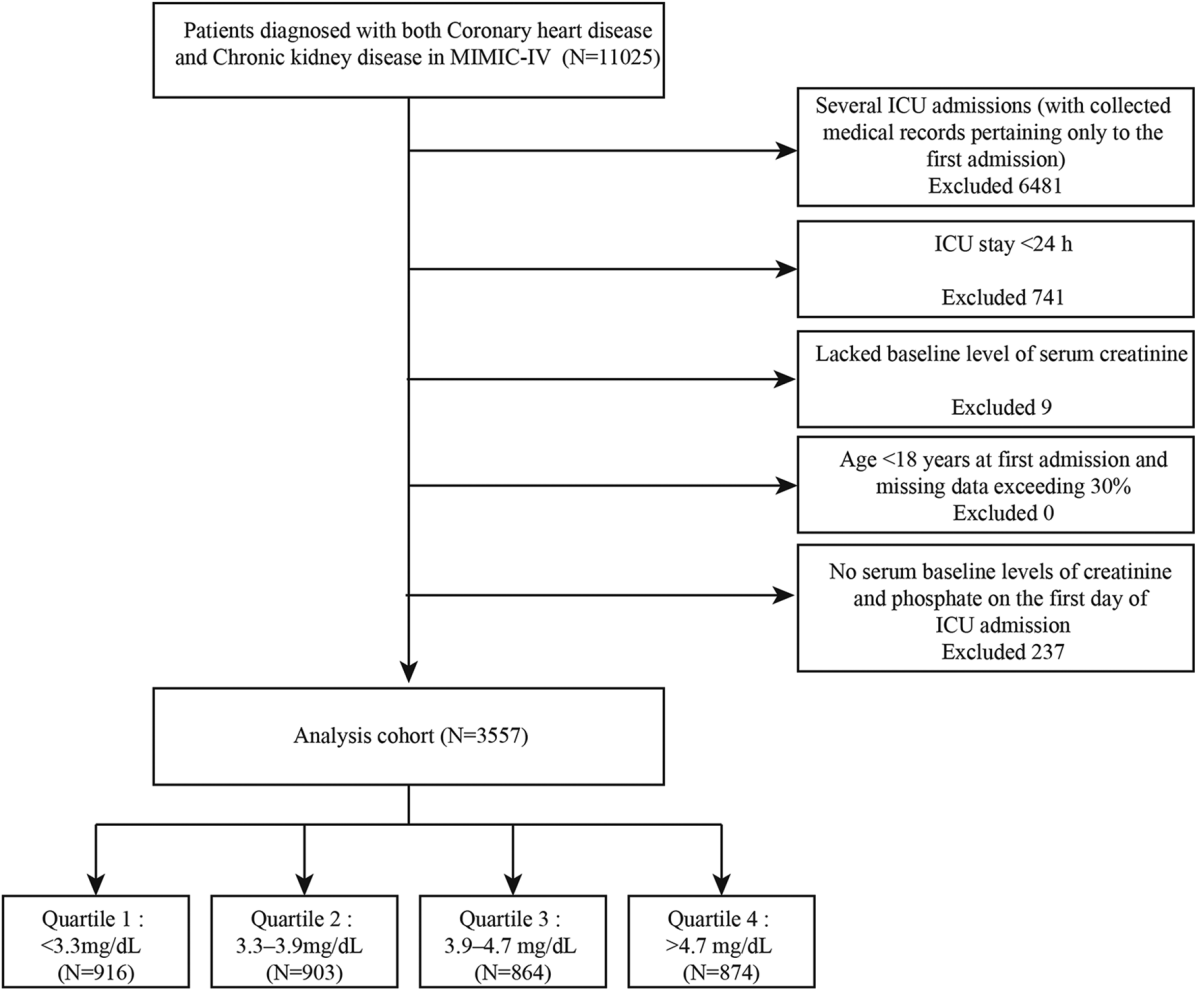
Flow chart of patient collection.

The cohort population included 3,557 patients from the MIMIC-IV with both CHD and CKD diagnoses. All patients were diagnosed per the International Classification of Diseases, 9th (ICD-9) and 10th (ICD-10) revisions. The criteria below were used to exclude ineligible patients: (1) several ICU admissions (with collected medical records pertaining only to the first admission), (2) ICU stay <24 h, (3) age <18 years at first admission, (4) no serum baseline levels of creatinine and phosphate on the first day of ICU admission, or (5) missing data exceeding 30%. Furthermore, Upon first admission to the ICU, the quartiles of serum phosphate levels were used to classify all patients into four distinct groups. Figure 1 presents the complete details concerning the inclusion of the study patients.

The MIMIC-IV database was searched to obtain patient information, such as demographics, laboratory test results, comorbidities, vital signs, and severity of illness scores, collected during the initial ICU admission using Structured Query Language (SQL) in PostgreSQL (version 16.0) and Navicat Premium (version 16.1) software. The extracted data were as follows: (1) demographics: gender, age, weight, height, and body mass index; (2) vital signs: diastolic blood pressure (DBP), mean blood pressure, systolic blood pressure (SBP), and heart rate; (3) laboratory tests: hemoglobin, blood urea nitrogen (BUN), platelet, hematocrit, estimated glomerular ﬁltration rate [eGFR; calculated using the formula for Modification of Diet in Renal Disease (13)], white blood cell (WBC), bicarbonate, serum phosphate, red blood cell (RBC), serum chloride, serum sodium, serum potassium, anion gap, prothrombin time (PT), serum calcium, international normalized ratio (INR), serum creatinine, partial thromboplastin time (PTT), and urine output; (4) comorbidities: acute kidney injury (AKI), peripheral vascular disease, atrial fibrillation (AF), diabetes mellitus (DM), rheumatic disease, congestive heart failure (CHF), dyslipidemia, liver disease, hypertension, AMI, chronic pulmonary disease, respiratory failure, and mechanical ventilation, all of which were diagnosed utilizing the combination of the ICD-9 and ICD-10 codes; and (5) severity of illness scores: the sequential organ failure assessment (SOFA) score, the acute physiology score III (APS III), the simplified acute physiology score II (SAPS II), the Oxford acute severity of illness score (OASIS), and the logistic organ dysfunction system (LODS) score. The missing data was filled up using multiple imputations.

#### 2.1.3 Clinical outcomes

The primary outcomes comprised the length of ICU and hospital stays as well as vital status during the short-term follow-up times of 30 and 90 days. ICU and hospital all-cause mortality were the secondary outcomes.

#### 2.1.4 Statistical analysis

The collected data were analyzed using descriptive statistics, with categorical variables presented as measures of quantity and frequency (%), and continuous variables presented as median with interquartile range or mean with standard deviation. Notably, the Shapiro–Wilk test was applied to establish the normality of continuous data. Subsequently, for non-normally distributed continuous data, we used the Wilcoxon rank-sum or Kruskal–Wallis tests. For those with normal distribution, we used *t*-tests or ANOVA. Fisher's exact tests or Pearson *χ*^2^ tests were utilized to compare categorical data. The Cox proportional hazards models were employed in the calculation of 95% confidence intervals (CIs) and hazard ratios (HRs) for serum phosphate and the secondary outcomes, along with adjustments for certain variables. Confounding factors with a variance inflation factor of ≥5 were excluded to eliminate model overfitting caused by multicollinearity among the variables. The multivariate model approach considered only clinically relevant factors linked with prognosis, yielding three models: an unadjusted model; a partially adjusted model including gender and age; and a fully adjusted model incorporating gender, age, SBP, cerebrovascular disease, DM, mechanical ventilation, respiratory failure, BUN, urine output, glucose, PTT, LODS score, and OASIS. The quartile levels were used to evaluate trends of *P*-values. The occurrence rate of the primary outcomes among the groups categorized based on the distinct serum phosphate levels was determined via Kaplan–Meier (KM) survival analysis. Between-group variance was evaluated via log-rank tests. The associations of serum phosphate levels with HRs were depicted using restricted cubic spline (RCS) regression models with a setting of five knots. The specificity and sensitivity of serum phosphate levels were examined using receiver operating characteristic curves and area under the curves (AUCs). Additional subgroup analysis was performed to determine the predictive value of serum phosphate for the secondary endpoints. The subgroups that were assessed were as follows: gender, age (<65 and >65 years), AKI, AF, CHF, DM, hypertension, and AMI. Statistical analyses were performed using R software (version 4.3.1). A double-sided *P*-value of <0.05 has been accepted as statistically significant.

### 2.2 Results

#### 2.2.1 Baseline characteristics

Table 1 presents the basic features of the patients categorized based on the quartiles of serum phosphate levels. A total of 3,557 patients [average age, 77.10 (69.0–84.2) years] were included, of which 1,152 (32.4%) were female. The mean serum phosphate level of the enrolled patients was 3.9 (3.3–4.7) mg/dl. Additionally, the hospital and ICU all-cause mortality rates were 14.6% and 10.0%, separately. Specifically, patients were divided based on the following quartiles: quartile 1 (Q1): <3.3 mg/dl, quartile 2 (Q2): 3.3–3.9 mg/dl, quartile 3 (Q3): 3.9–4.7 mg/dl, and quartile 4 (Q4): >4.7 mg/dl. The mean serum phosphate levels of these four groups were 2.9 (2.6–3.1), 3.6 (3.5–3.8), 4.2 (4.1–4.4), and 5.6 (5.1–6.5) mg/dl, respectively.

**Table 1:** Baseline characteristics of study populations based on the serum phosphate quartiles.

Categories	Total	Q1 (<3.3 mg/dl)	Q2 (3.3–3.9 mg/dl)	Q3 (3.9–4.7 mg/dl)	Q4 (>4.7 mg/dl)	*P*
*N*	3,557	916	903	864	874	* *
Gender						
Female	1,152 (32.4%)	265 (28.9%)	304 (33.7%)	295 (34.1%)	288 (33%)	0.071
Male	2,405 (67.6%)	651 (71.1%)	599 (66.3%)	569 (65.9%)	586 (67%)	
Age, years	77.1 (69.0–84.2)	78.7 (69.9–85.2)	78.1 (70.4–85.0)	76.5 (68.2–83.7)	74.5 (66.7–82.8)	<.001
Height, cm	170.0 (160.0–178.0)	170.0 (160.0–178.0)	168.0 (160.0–178.0)	169.0 (160.0–178.0)	168.0 (160.0–178.0)	0.516
Weight, kg	80.6 (68.5–95.2)	80.0 (68.0–94.5)	80.5 (69.5–94.1)	81.9 (69.0–96.3)	80.0 (67.0–96.0)	0.274
BMI, kg/m^2^	28.3 (25.1–32.3)	27.9 (24.9–31.8)	28.4 (25.1–32.2)	28.7 (25.5–33.0)	28.1 (24.8–32.2)	0.029
SBP, mmHg	115.7 (106.5–127.5)	117.1 (108.2–129.5)	115.6 (107.0–126.3)	116.6 (107.1–129.9)	112.9 (104.1–125.3)	<.001
DBP, mmHg	57.8 (51.6–65.0)	58.5 (52.3–65.5)	57.0 (51.0–64.7)	57.5 (51.4–64.7)	57.8 (51.6–65.0)	0.019
MBP, mmHg	74.2 (68.6–80.6)	75.1 (69.0–81.4)	73.8 (68.7–79.9)	74.3 (68.3–80.4)	73.3 (67.5–80.5)	0.023
Heart rate, bpm	79.2 (70.4–88.8)	79.1 (70.8–88.6)	78.9 (70.3–87.6)	79.0 (70.5–89.0)	79.7 (70.1–90.0)	0.497
Comorbidities, *n* (%)						
AKI	947 (26.6%)	186 (20.3%)	200 (22.1%)	222 (25.7%)	339 (38.8%)	<.001
AF	1,633 (45.9%)	408 (44.5%)	425 (47.1%)	380 (44%)	420 (48.1%)	0.252
Chronic pulmonary disease	1,121 (31.5%)	279 (30.5%)	286 (31.7%)	276 (31.9%)	280 (32%)	0.881
Cerebrovascular disease	565 (15.9%)	155 (16.9%)	152 (16.8%)	144 (16.7%)	114 (13%)	0.071
CHF	2,303 (64.7%)	492 (53.7%)	591 (65.4%)	589 (68.2%)	631 (72.2%)	<.001
DM	1,981 (55.7%)	452 (49.3%)	483 (53.5%)	530 (61.3%)	516 (59%)	<.001
Dyslipidemia	1,064 (29.9%)	253 (27.6%)	272 (30.1%)	282 (32.6%)	257 (29.4%)	0.14
Liver disease	295 (8.3%)	56 (6.1%)	57 (6.3%)	69 (8%)	113 (12.9%)	<.001
Hypertension	3,278 (92.2%)	843 (92%)	841 (93.1%)	791 (91.6%)	803 (91.9%)	0.627
Mechanical ventilation	1,268 (35.6%)	302 (33%)	331 (36.7%)	283 (32.8%)	352 (40.3%)	0.002
AMI	1,854 (52.1%)	440 (48%)	465 (51.5%)	468 (54.2%)	481 (55%)	0.014
Peripheral vascular disease	866 (24.3%)	214 (23.4%)	221 (24.5%)	219 (25.3%)	212 (24.3%)	0.81
Respiratory failure	1,010 (28.4%)	203 (22.2%)	200 (22.1%)	253 (29.3%)	354 (40.5%)	<.001
Rheumatic disease	144 (4%)	40 (4.4%)	41 (4.5%)	25 (2.9%)	38 (4.3%)	0.265
Laboratory tests						
RBC, #/ul	3.4 (3.0–3.8)	3.4 (3.0–3.9)	3.4 (3.0–3.9)	3.3 (2.9–3.8)	3.2 (2.9–3.7)	<.001
WBC, K/ul	10.8 (8.2–14.6)	10.7 (8.0–13.9)	10.8 (8.0–14.4)	10.7 (8.4–14.1)	11.2 (8.4–15.8)	0.001
Platelets, K/ul	178.5 (135.5–237.5)	166.8 (131.0–219.8)	177.0 (133.5–230.2)	184.5 (142.0–241.8)	190.2 (136.5–256.0)	<.001
Hematocrit, %	30.1 (27.0–34.4)	30.6 (27.7–35.0)	30.4 (27.3–34.8)	30.0 (26.7–34.0)	29.6 (26.2–33.4)	<.001
Hemoglobin, g/dl	9.8 (8.7–11.2)	10.1 (9.0–11.6)	10.0 (8.8–11.4)	9.8 (8.6–11.0)	9.5 (8.4–10.8)	<.001
eGFR, ml/min/1.73 m^2^	48.1 (30.8–65.5)	58.9 (47.0–75.7)	54.0 (39.5–68.6)	44.4 (30.3–58.9)	28.3 (16.7–46.8)	<.001
Serum creatinine, mg/dl	1.4 (1.1–2.0)	1.1 (1.0–1.4)	1.3 (1.0–1.6)	1.5 (1.2–2.1)	2.2 (1.4–3.5)	<.001
Anion gap, mmol/L	15.5 (13.0–18.0)	14.0 (12.5–16.5)	14.5 (12.5–16.5)	15.5 (13.5–18.0)	19.0 (16.0–22.0)	<.001
Serum bicarbonate, mEq/L	22.5 (20.0–25.0)	23.0 (20.5–25.0)	23.0 (21.0–25.0)	22.5 (20.2–25.0)	21.0 (18.0–24.0)	<.001
BUN, mg/dl	37.0 (25.5–56.0)	28.0 (20.5–38.5)	31.5 (23.0–43.5)	39.5 (28.0–57.2)	59.5 (41.5–82.0)	<.001
Urine Output, ml/24 h	1,320.0 (727.0–2,115.0)	1,445.0 (940.0–2,250.0)	1,420.0 (909.0–2,100.0)	1,406.5 (838.5–2,252.5)	881.0 (275.0–1,669.0)	<.001
Serum calcium, mg/dl	8.4 (8.1–8.9)	8.4 (8.0–8.8)	8.5 (8.1–8.9)	8.6 (8.1–9.0)	8.4 (7.9–8.9)	<.001
Serum chloride, mEq/L	103.5 (99.0–107.0)	105.5 (101.5–108.2)	104.5 (100.2–107.5)	103.0 (99.0–107.0)	100.5 (95.5–105.0)	<.001
Glucose, mg/dl	136.5 (111.5–178.0)	130.5 (110.0–169.0)	135.0 (112.2–172.0)	138.0 (112.0–181.5)	145.5 (112.5–189.5)	<.001
Serum sodium, mEq/L	138.5 (135.5–141.0)	139.0 (136.5–141.0)	138.5 (136.0–140.5)	138.5 (135.8–141.0)	137.5 (134.5–140.0)	<.001
Serum potassium, mEq/L	4.4 (4.0–4.9)	4.2 (3.9–4.6)	4.3 (4.0–4.7)	4.5 (4.1–4.9)	4.8 (4.2–5.2)	<.001
INR	1.3 (1.1–1.5)	1.3 (1.1–1.5)	1.2 (1.1–1.4)	1.2 (1.1–1.5)	1.3 (1.1–1.7)	<.001
PT	14.2 (12.6–16.6)	14.2 (12.7–16.4)	13.8 (12.6–16.0)	14.1 (12.6–16.1)	14.6 (12.8–18.6)	<.001
APTT	33.5 (28.6–47.0)	32.0 (28.1–42.1)	33.1 (28.4–45.3)	33.4 (28.2–47.2)	37.1 (29.8–54.5)	<.001
Serum phosphate, mg/dl	3.9 (3.3–4.7)	2.9 (2.6–3.1)	3.6 (3.5–3.8)	4.2 (4.1–4.4)	5.6 (5.1–6.5)	<.001
APS III	49.0 (40.0–61.0)	44.0 (36.0–55.0)	45.0 (38.0–56.0)	50.0 (41.0–60.0)	58.0 (49.0–70.0)	<.001
SOFA	6.0 (4.0–8.0)	5.0 (3.0–7.0)	5.0 (3.0–7.0)	5.0 (3.0–7.0)	7.0 (5.0–10.0)	<.001
LODS	5.0 (4.0–7.0)	5.0 (3.0–6.0)	5.0 (4.0–7.0)	5.0 (4.0–7.0)	7.0 (5.0–9.0)	<.001
OASIS	32.0 (26.0–38.0)	31.0 (26.0–37.0)	31.0 (26.0–37.0)	31.0 (25.0–37.0)	34.0 (28.0–41.0)	<.001
SAPS II	41.0 (35.0–50.0)	39.0 (32.0–46.0)	40.0 (34.0–47.5)	41.0 (35.0–48.0)	47.0 (39.0–56.0)	<.001
Events						
Hospital expire flag, *n* (%)	520 (14.6%)	109 (11.9%)	84 (9.3%)	106 (12.3%)	221 (25.3%)	<.001
LOS hospital, days	8.7 (5.6–13.9)	8.1 (5.5–12.8)	8.8 (5.5–13.6)	9.0 (5.7–14.0)	9.6 (5.8–15.0)	0.001
ICU expire flag, *n* (%)	357 (10%)	63 (6.9%)	55 (6.1%)	76 (8.8%)	163 (18.6%)	<.001
LOS ICU, days	2.7 (1.7–4.6)	2.4 (1.5–4.3)	2.4 (1.6–4.1)	2.6 (1.6–4.2)	3.2 (1.9–5.9)	<.001

BMI, body mass index; SBP, systolic blood pressure; DBP, diastolic blood pressure; MBP, mean blood pressure; AKI, acute kidney injury; AF, atrial fibrillation; CHF, congestive heart failure; DM, diabetes mellitus; AMI, acute myocardial infarction; eGFR, estimated glomerular ﬁltration rate; RBC, red blood cell; WBC, white blood cell; BUN, blood urea nitrogen; INR, international normalized ratio; PT, prothrombin time; APTT, activated partial thromboplastin time; APS III, acute physiology score III; SOFA, sequential organ failure assessment; LODS; OASIS, oxford acute severity of illness score; SAPS II, simplified acute physiological score II; LOS hospital, length of stay hospital; LOS ICU, length of stay intensive care unit.

Compared with the groups with lower serum phosphate levels, those with increased concentrations were associated with younger age; higher prevalence rate of DM, CHF, liver disease, respiratory failure, mechanical ventilation, AKI, and AMI; higher severity of illness scores; higher levels of WBC, platelets, anion gap, BUN, serum calcium, glucose, serum potassium, INR, PT, PTT, and serum creatinine; and lower values of SBP, RBC, hematocrit, hemoglobin, eGFR, bicarbonate, urine output, serum chloride, and serum sodium. Moreover, the increasing serum phosphate levels from Q1 to Q4 were correlated with extended duration of stay in the ICU (2.4 days vs. 2.4 days vs. 2.6 days vs. 3.2 days) and hospital (8.1 days vs. 8.8 days vs. 9.0 days vs. 9.6 days) as well as higher all-cause mortality in the ICU (6.9% vs. 6.1% vs. 8.8% vs. 18.6%) and hospital (11.9% vs. 9.3% vs. 12.3% vs. 25.3%), with *P* < 0.001 for all.

The baseline characteristics of the patients who survived for 30 days (30-day survivor group) compared with those who did not survive for 30 days (30-day non-survivor group) are displayed in Table 2. The 30-day non-survivor group exhibited older age and elevated heart rate levels in comparison to the 30-day survivor group, along with a higher prevalence of AKI, AF, cerebrovascular disease, CHF, liver disease, mechanical ventilation, AMI, and respiratory failure (all *P* < 0.05). In the case of the laboratory test results, the 30-day non-survivor group exhibited higher levels of WBC, BUN, anion gap, glucose, serum potassium, PTT, PT, INR, and serum creatinine, and lower values of eGFR, serum bicarbonate, urine output, and serum chloride in comparison to the 30-day survivors (all *P* < 0.05). Nevertheless, gender, DBP, dyslipidemia, rheumatic disease, platelets, hematocrit, hemoglobin, serum calcium, serum sodium, and serum potassium weren't different significantly (all *P* > 0.05). Regarding the severity of illness scores, the 30-day non-survivors recorded higher APS III, LODS score, SOFA score, OASIS, and SAPS II than those of the 30-day survivors. Furthermore, 30-day non-survivors had remarkably higher serum phosphate levels in comparison to 30-day survivors [4.3 (3.5–5.3) mg/dl vs. 3.8 (3.3–4.5) mg/dl, *P* < 0.001]. In the 90-day survivor and non-survivor groups, similar differences were noticed (Supplementary Table S1).

**Table 2:** Baseline characteristics of the patients grouped into 30-day non-survivor and survivor groups.

Categories	Total	Non-survivors	Survivors	*P*
*N*	3,557	665	2,892	
Gender				
Female	1,152 (32.4%)	217 (32.6%)	935 (32.3%)	.917
Male	2,405 (67.6%)	448 (67.4%)	1,957 (67.7%)	
Age, years	77.1 (69.0–84.2)	81.7 (73.4–87.6)	76.1 (68.1–83.3)	<.001
Height, cm	170.0 (160.0–178.0)	168.0 (158.0–178.0)	170.0 (160.0–178.0)	.028
Weight, kg	80.6 (68.5–95.2)	76.2 (65.5–91.0)	81.6 (69.2–96.2)	<.001
BMI, kg/m^2^	28.3 (25.1–32.3)	27.2 (24.0–31.2)	28.6 (25.4–32.6)	<.001
SBP, mmHg	115.7 (106.5–127.5)	110.5 (102.8–121.9)	116.9 (107.5–129.1)	<.001
DBP, mmHg	57.8 (51.6–65.0)	58.5 (51.4–64.9)	57.6 (51.7–65.0)	.702
MBP, mmHg	74.2 (68.6–80.6)	73.0 (67.3–79.5)	74.5 (68.8–80.8)	<.001
Heart rate, bpm	79.2 (70.4–88.8)	83.3 (70.9–93.9)	78.7 (70.3–87.7)	<.001
Comorbidities, *n* (%)				
AKI	947 (26.6%)	264 (39.7%)	683 (23.6%)	<.001
AF	1,633 (45.9%)	345 (51.9%)	1,288 (44.5%)	<.001
Chronic pulmonary disease	1,121 (31.5%)	222 (33.4%)	899 (31.1%)	.270
Cerebrovascular disease	565 (15.9%)	131 (19.7%)	434 (15%)	.003
CHF	2,303 (64.7%)	477 (71.7%)	1,826 (63.1%)	<.001
DM	1,981 (55.7%)	334 (50.2%)	1,647 (57%)	.002
Dyslipidemia	1,064 (29.9%)	188 (28.3%)	876 (30.3%)	.328
Liver disease	295 (8.3%)	91 (13.7%)	204 (7.1%)	<.001
Hypertension	3,278 (92.2%)	600 (90.2%)	2,678 (92.6%)	.048
Mechanical ventilation	1,268 (35.6%)	284 (42.7%)	984 (34%)	<.001
AMI	1,854 (52.1%)	393 (59.1%)	1,461 (50.5%)	<.001
Peripheral vascular disease	866 (24.3%)	174 (26.2%)	692 (23.9%)	.245
Respiratory failure	1,010 (28.4%)	348 (52.3%)	662 (22.9%)	<.001
Rheumatic disease	144 (4%)	31 (4.7%)	113 (3.9%)	.435
Laboratory tests				
RBC, #/ul	3.4 (3.0–3.8)	3.3 (2.9–3.8)	3.4 (3.0–3.8)	.149
WBC, K/ul	10.8 (8.2–14.6)	12.0 (8.8–16.6)	10.7 (8.1–14.2)	<.001
Platelets, K/ul	178.5 (135.5–237.5)	183.0 (128.5–253.5)	178.0 (136.0–234.5)	.421
Hematocrit, %	30.1 (27.0–34.4)	30.7 (27.2–35.0)	30.0 (27.0–34.3)	.026
Hemoglobin, g/dl	9.8 (8.7–11.2)	9.8 (8.6–11.2)	9.8 (8.8–11.2)	.706
eGFR, ml/min/1.73 m^2^	48.1 (30.8–65.5)	40.3 (24.8–57.8)	50.0 (32.9–67.4)	<.001
Serum creatinine, mg/dl	1.4 (1.1–2.0)	1.6 (1.2–2.4)	1.3 (1.0–1.9)	<.001
Anion gap, mmol/L	15.5 (13.0–18.0)	17.5 (15.5–21.0)	15.0 (13.0–17.5)	<.001
Serum bicarbonate, mEq/L	22.5 (20.0–25.0)	21.0 (18.0–24.5)	22.5 (20.5–25.0)	<.001
BUN, mg/dl	37.0 (25.5–56.0)	49.0 (33.5–71.0)	34.5 (24.0–52.2)	<.001
Urine output, ml/24 h	1,320.0 (727.0–2,115.0)	880.0 (342.0–1,492.0)	1,415.0 (850.0–2,240.0)	<.001
Serum calcium, mg/dl	8.4 (8.1–8.9)	8.4 (8.0–8.9)	8.5 (8.1–8.9)	.068
Serum chloride, mEq/L	103.5 (99.0–107.0)	102.0 (97.5–106.0)	104.0 (99.5–107.5)	<.001
Glucose, mg/dl	136.5 (111.5–178.0)	153.5 (120.5–198.5)	133.0 (110.5–172.0)	<.001
Serum sodium, mEq/L	138.5 (135.5–141.0)	138.0 (135.0–141.0)	138.5 (136.0–140.5)	.540
Serum potassium, mEq/L	4.4 (4.0–4.9)	4.4 (4.1–5.0)	4.4 (4.0–4.8)	.006
INR	1.3 (1.1–1.5)	1.4 (1.2–1.9)	1.2 (1.1–1.4)	<.001
PT	14.2 (12.6–16.6)	15.3 (13.2–20.6)	13.9 (12.6–16.0)	<.001
PTT	33.5 (28.6–47.0)	37.1 (29.6–58.0)	33.0 (28.4–45.3)	<.001
Serum phosphate, mg/dl	3.9 (3.3–4.7)	4.3 (3.5–5.3)	3.8 (3.3–4.5)	<.001
APS III	49.0 (40.0–61.0)	61.0 (50.0–77.0)	47.0 (39.0–58.0)	<.001
SOFA	6.0 (4.0–8.0)	7.0 (5.0–10.0)	5.0 (3.0–7.0)	<.001
LODS	5.0 (4.0–7.0)	7.0 (5.0–9.0)	5.0 (4.0–7.0)	<.001
OASIS	32.0 (26.0–38.0)	37.0 (31.0–44.0)	31.0 (26.0–36.0)	<.001
SAPS II	41.0 (35.0–50.0)	49.0 (41.0–59.0)	40.0 (33.0–47.0)	<.001

BMI, body mass index; SBP, systolic blood pressure; DBP, diastolic blood pressure; MBP, mean blood pressure; AKI, acute kidney injury; AF, atrial fibrillation; CHF, congestive heart failure; DM, diabetes mellitus; AMI, acute myocardial infarction; eGFR, estimated glomerular ﬁltration rate; RBC, red blood cell; WBC, white blood cell; BUN, blood urea nitrogen; INR, international normalized ratio; PT, prothrombin time; APTT, activated partial thromboplastin time; APS III, acute physiology score III; SOFA, sequential organ failure assessment; LODS; OASIS, oxford acute severity of illness score; SAPS II, simplified acute physiological score II.

#### 2.2.2 Primary outcomes

**Figure 2:**
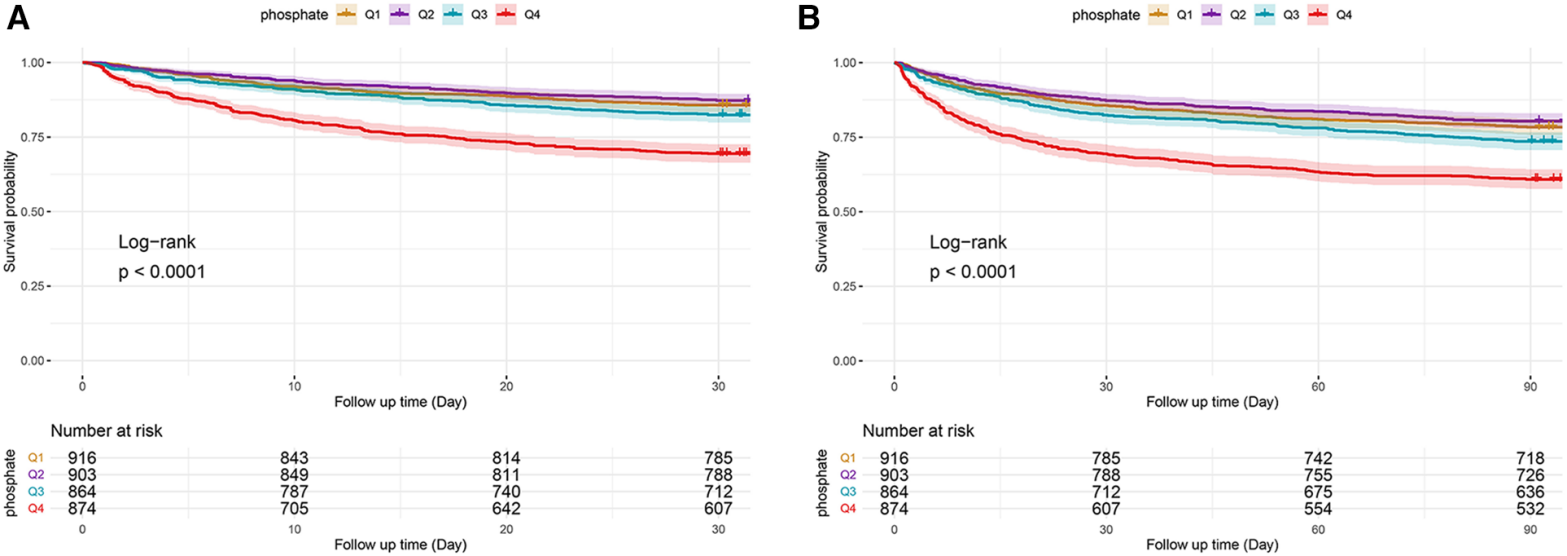
Cumulative survival rate as shown by Kaplan-Meier curves by serum phosphate quartile groups at the follow-up times of 30 days (**A**) and 90 days (**B**). Quartiles: Q1: (<3.3); Q2: (3.3–3.9); Q3: (3.9–4.7); Q4: (>4.7).

Figure 2 depicts the KM survival curve analysis for the occurrence of primary outcomes among the quartile groups. Patient mortality risk was higher in those with elevated serum phosphate levels during the follow-up periods of 30 and 90 days (all log-rank *P* < 0.001).

**Figure 3:**
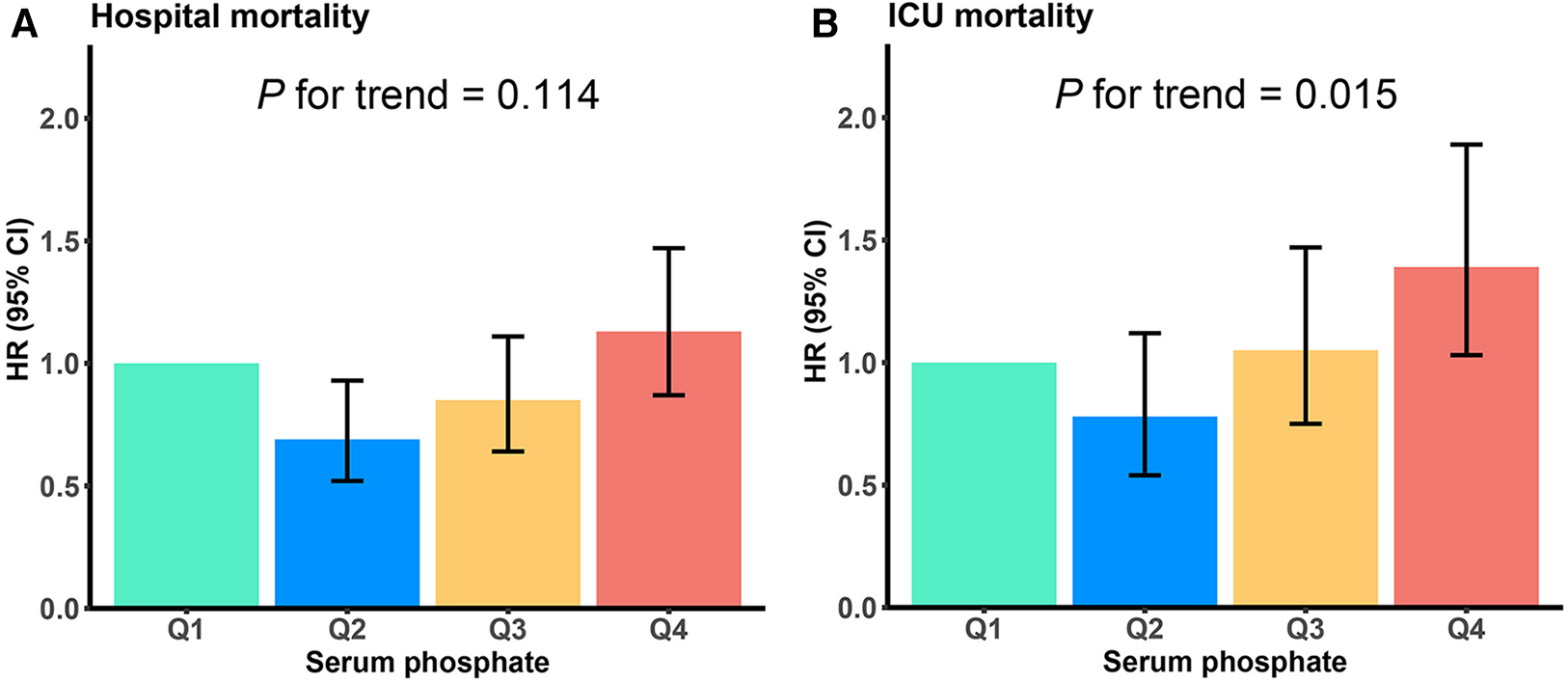
Hospital (**A**) and ICU (**B**) mortality risk based on the following quartiles of serum phosphate levels. Q1 is the reference quartile, and the error bars indicate 95% CI. HR, hazard ratio; CI, confidence intervals.

Serum phosphate was shown to be substantially linked to death risk from all causes in the hospital, as determined by the Cox proportional hazards analysis. This correlation was observed in all three models when it was regarded as a continuous variable, i.e., the unadjusted [HR, 1.30 (95% CI: 1.24–1.36)], partially adjusted [HR, 1.36 (95% CI: 1.30–1.44)], and fully adjusted [HR, 1.10 (95% CI: 1.03–1.18)] models (all *P* < 0.05). Moreover, patients in the higher quartiles had an increasing tendency in serum phosphate levels when compared to those in the lowest quartile and were related to an elevated hospital mortality risk in the unadjusted [Q1 vs. Q4: HR, 2.37 (95% CI: 1.88–2.98)] and partially adjusted [Q1 vs. Q4: HR, 2.65 (95% CI: 2.11–3.34)] models (both *P* < 0.001; both *P* for trend <0.001), with serum phosphate levels as a nominal variable (Table 3 and Figure 3A). The analysis of the ICU death risk revealed similar results in the unadjusted [Q1 vs. Q4: HR, 2.96 (95% CI: 2.22–3.97)], partially adjusted [Q1 vs. Q4: HR, 3.22 (95% CI: 2.41–4.32)], and fully adjusted [Q1 vs. Q4: HR, 1.39 (95% CI: 1.03–1.89)] models (all *P* < 0.05; all *P* for trend <0.05) (Table 3 and Figure 3B).

**Table 3:** Cox proportional hazard ratios (HRs) for death risk.

Categories	Model 1			Model 2			Model 3		
	HR	*P*-value	*P* for trend	HR	*P*-value	*P* for trend	HR	*P*-value	*P* for trend
Hospital									
Continuous variable	1.30 (1.24–1.36)	*P *< .001		1.36 (1.30–1.44)	*P *< .001		1.10 (1.03–1.18)	*P *= .007	
Quartile			<0.001			<0.001			0.114
Q1 (<3.3)	Reference			Reference			Reference		
Q2 (3.3–3.9)	0.77 (0.58–1.03)	*P *= .077		0.77 (0.58–1.03)	*P *= .078		0.69 (0.52–0.93)	*P *= .013	
Q3 (3.9–4.7)	1.05 (0.80–1.37)	*P *= .729		1.10 (0.84–1.44)	*P *= .488		0.85 (0.64–1.11)	*P *= .230	
Q4 (>4.7)	2.37 (1.88–2.98)	*P *< .001		2.65 (2.11–3.34)	*P *< .001		1.13 (0.87–1.47)	*P *= .350	
ICU									
Continuous variable	1.35 (1.27–1.42)	*P *< .001		1.40 (1.32–1.48)	*P *< .001		1.14 (1.07–1.22)	*P *< .001	
Quartile			<0.001			<0.001			0.015
Q1 (<3.3)	Reference			Reference			Reference		
Q2 (3.3–3.9)	0.88 (0.61–1.26)	*P *= .483		0.88 (0.61–1.26)	*P *= .486		0.78 (0.54–1.12)	*P *= .170	
Q3 (3.9–4.7)	1.30 (0.93–1.81)	*P *= .126		1.35 (0.96–1.88)	*P *= .083		1.05 (0.75–1.47)	*P *= .793	
Q4 (>4.7)	2.96 (2.22–3.97)	*P* < .001		3.22 (2.41–4.32)	*P *< .001		1.39 (1.03–1.89)	*P *= .033	

Model 1: unadjusted.

Model 2: adjusted for gender, age.

Model 3: adjusted for gender, age, SBP, cerebrovascular disease, DM, mechanical ventilation, respiratory failure, BUN, urine output, glucose, APTT, LODS score, OASIS.

**Figure 4:**
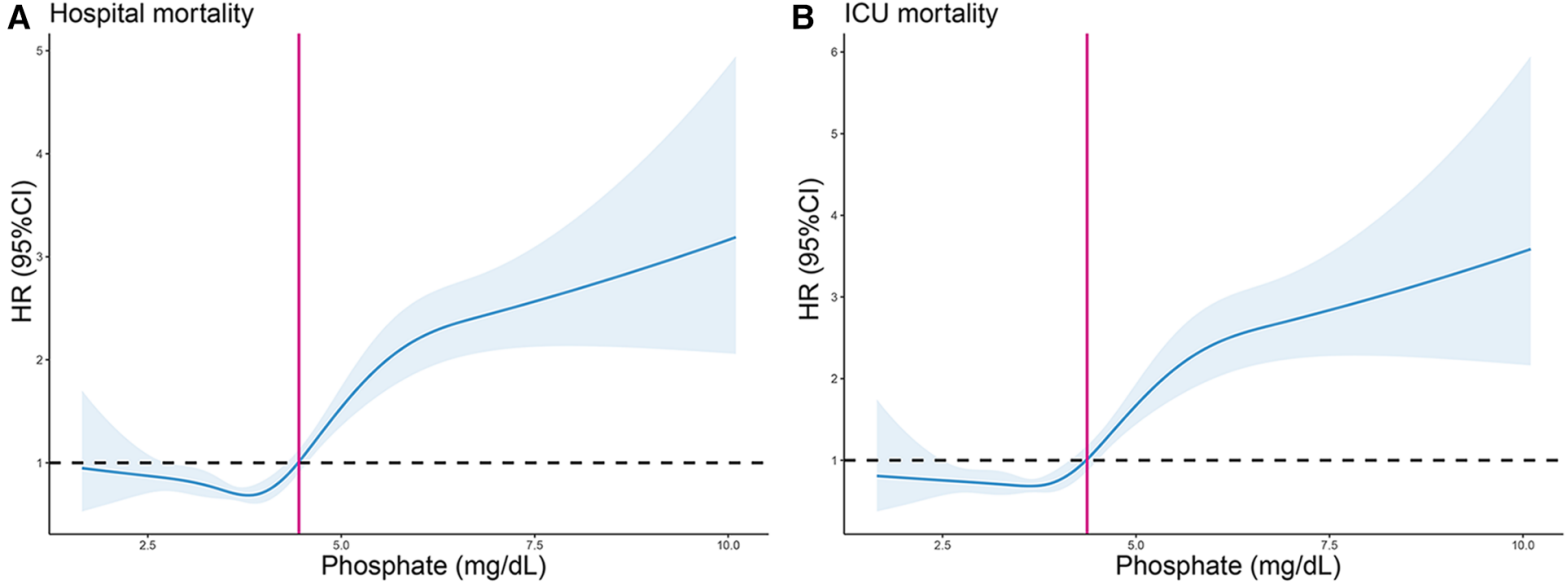
Restricted cubic spline (RCS) curve for the HR of serum phosphate for predicting hospital (**A**) and ICU (**B**) all-cause mortality. Vertical lines signify the serum phosphate values of 4.45 and 4.37 mg/dl in the hospital and ICU, respectively. HR, hazard ratio; CI, confidence interval.

**Figure 5:**
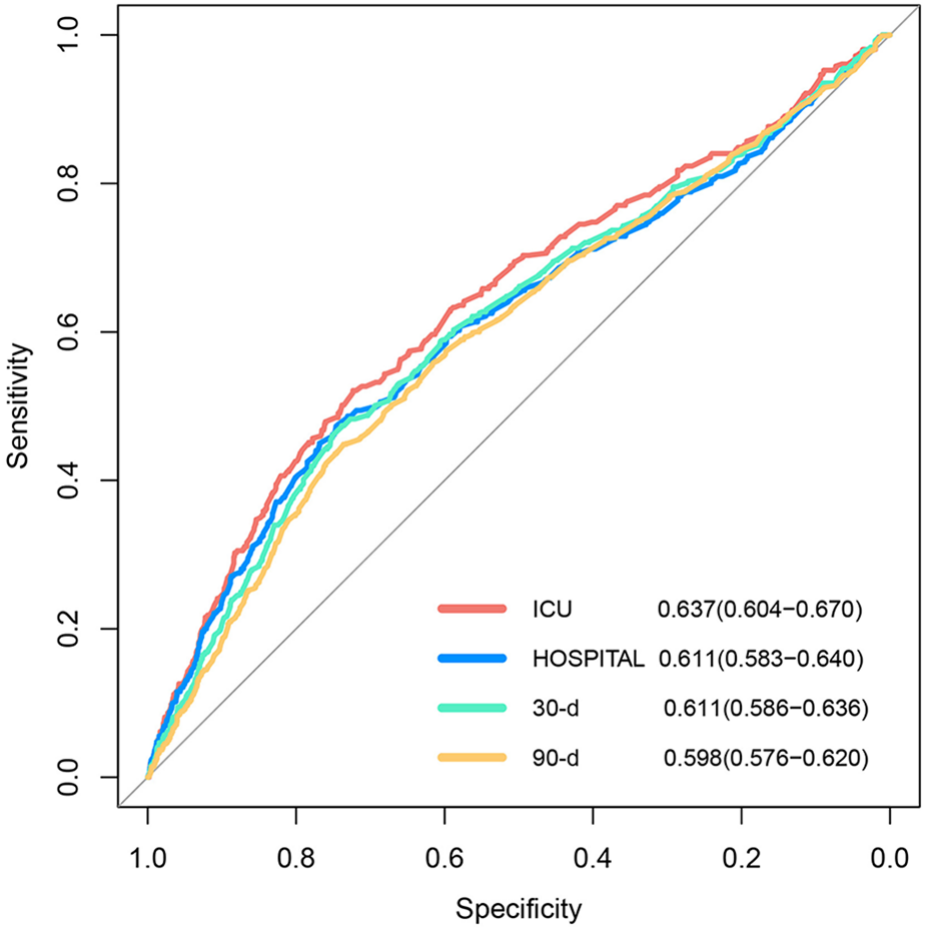
Receiver operating curves of ICU and hospital and during the 30- and 90-day follow-ups, with areas under the curve of 0.637, 0.611, 0.611, and 0.598, respectively.

As demonstrated in Figure 4, the HRs exhibited an upward trend when the serum phosphate levels surpassed the values of 4.37 and 4.45 mg/dl in the ICU and hospital, respectively. Conversely, the HRs of the serum phosphate values lower than these specified thresholds remained relatively stable. Furthermore, the RCS regression analysis demonstrated that the ICU and hospital death risk was strongly nonlinearly correlated with serum phosphate levels (both *P* for non-linearity <0.001). Lastly, the AUCs of the serum phosphate levels for predicting death risk in the ICU and hospital and during the follow-ups at 30 and 90 days were 0.637 (95% CI: 0.604–0.670), 0.611 (0.583–0.640), 0.611 (0.586–0.636), and 0.598 (0.576–0.620), respectively (Figure 5).

#### 2.2.3 Subgroup analysis

**Figure 6:**
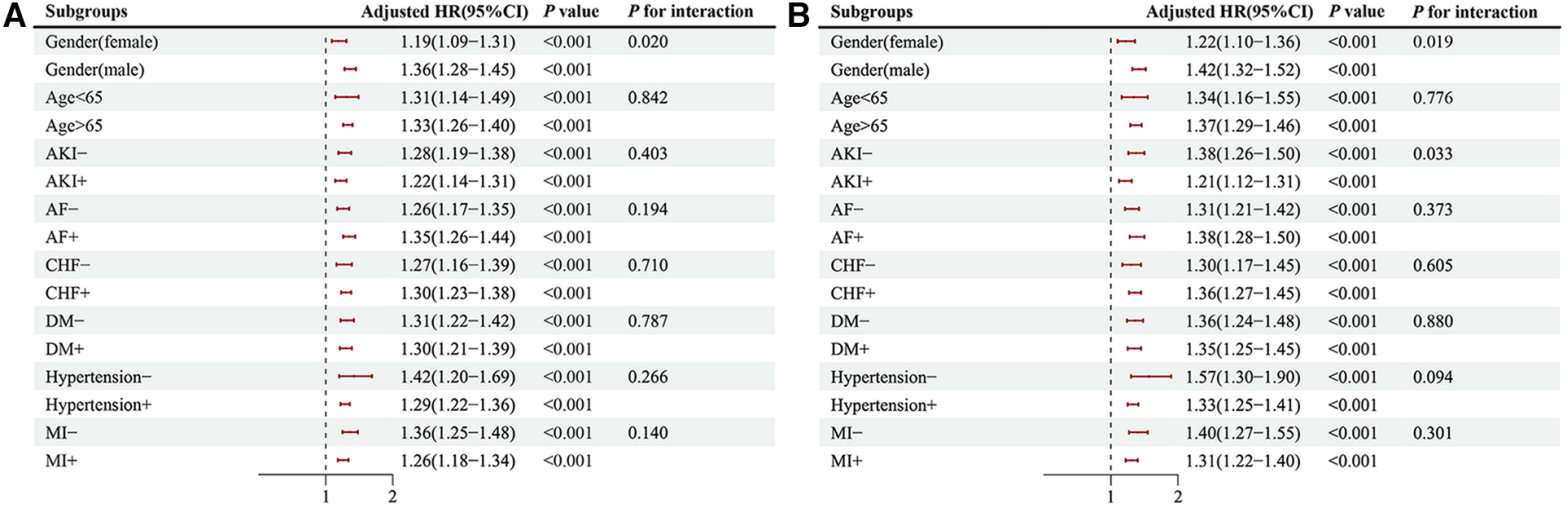
The primary endpoint subgroup forest plots, hospital (**A**), ICU (**B**). HR, hazard ratio; CI, confidence interval; AKI, acute kidney injury; AF, atrial fibrillation; CHF, congestive heart failure; DM, diabetes; AMI, acute myocardial infarct.

We further evaluated the risk stratification significance of the serum phosphate levels for mortality in the hospital and ICU according to the various subgroups of gender, age, AKI, AF, CHF, DM, hypertension, and AMI (Figure 6). In the case of hospital mortality, mortality differences were detected in only the gender subgroup [males: HR, 1.36 (95% CI: 1.28–1.45) vs. females: HR, 1.19 (95% CI: 1.09–1.31), *P* for interaction = 0.020] (Figure 6A). Further, in the case of ICU mortality showed differences in mortality risk within two subgroups, i.e., the gender subgroup [males: HR, 1.42 (95% CI: 1.32–1.52) vs. females: HR, 1.22 (95% CI: 1.10–1.36), *P* for interaction = 0.019], and AKI subgroup [patients with AKI: HR, 1.21 (95% CI: 1.12–1.31) vs. patients without AKI: HR, 1.38 (95% CI: 1.26–1.50), *P* for interaction = 0.033] (Figure 6B).

### 2.3 Discussion

For critically ill patients with CHD accompanied by CKD, this is the first research to utilize a large ICU database to elucidate the prognostic value of serum phosphate levels in predicting outcomes. Accordingly, the primary finding of our study was that elevated serum phosphate levels on ICU admission were linked to death risk in the ICU and hospital as well as during short-term follow-up in this patient population. Moreover, this trend persisted for ICU all-cause mortality even after adjusting for confounding risk variables. Lastly, our study results demonstrated that serum phosphate level exhibited a robust non-linear relationship with the ICU and hospital death risk among patients with CHD accompanied by CKD.

Morbidity and death rates in patients with CKD are notably influenced by CVD (3). Conversely, CKD is linked to numerous severe cardiovascular complications, including silent ischemia, serious cardiac arrhythmia, HF, and AMI, contributing to an elevated risk of all-cause mortality and CHD-related mortality (14, 15). Therefore, CHD and CKD can exacerbate each other. Moreover, timely prognostic evaluation is of crucial importance for critically ill patients having CHD concurrent with CKD. However, the widely used prognostic clinical markers have been suggested to be insufficient. Although serum phosphate is a readily accessible indicator, it is not frequently employed for evaluating this patient group. Additionally, most patients with CKD exhibit elevated serum phosphate levels, thereby indicating that they have a higher risk for CHD-related mortality (16, 17). Consequently, strategically managing serum phosphate concentrations within an optimal range may serve as a pivotal therapeutic approach for mitigating mortality risk in patients with such conditions.

Serum phosphate is used as an indicator of mineral metabolism and electrolyte disturbances and is widely employed in the ICU to assess the disease severity and progression of critically ill patients, particularly those with sepsis (18, 19). A previous study highlighted that critically ill patients present with elevated serum phosphate levels, which in turn linked to increased death risk (20). In a large-scale cohort investigation of community-living older men, heightened serum phosphate concentrations were strongly linked to death from CVD and all causes (21). Two longitudinal investigations have also shown that an elevated risk of HF and death was correlated with elevated serum phosphate levels, in patients who had experienced AMI (22, 23). In support of the previous findings, a multicenter, population-based prospective cohort study involving 14,709 participants discovered that serum phosphate was substantially linked to HF risk (24). High serum phosphate concentrations in patients with CKD have been linked by other researchers to vascular calcification (25, 26), arterial stiffness (27), incident cardiovascular events (CVEs) (22, 28, 29), and cardiovascular mortality (30). Another recent study indicated that increased serum phosphate levels and cardiovascular complications in population with CKD were significant risk factors for the early prediction of CVEs (31). A retrospective cohort study suggested that AMI risk was independently correlated with elevated serum phosphate levels among veterans with CKD (32). All these studies imply that serum phosphate is a promising prognostic indicator to predict clinical outcomes in patients with CHD accompanied by CKD. However, limited research has been conducted on the link between serum phosphate and hospital and ICU mortality from all causes in patients with CHD, CKD, or CHD combined with CKD during short-term follow-up.

Scarce data are currently available on the relationships between mortality and serum phosphate in critically ill populations. Zheng et al. recently found that serum phosphate serves as a disease severity indicator as well as an clinical marker for all-cause mortality in the ICU (20). Consistent with this prior result, we reported that serum phosphate was an effective clinical prognostic indicator for ICU all-cause mortality in patients with CHD combined with CKD. However, Zheng et al. indicated no significant increase in the duration of hospitalization for patients who had elevated serum phosphate levels (20), whereas our research showed that the higher quartiles of serum phosphate levels were correlated with extended stays in the ICU and hospital. This observation highlights the significance of optimizing clinical management to reduce adverse event incidences. Additionally, our study revealed a high prevalence of CVD and vascular health risk markers among the patients with elevated serum phosphate levels, with approximately 90% presenting with hypertension, nearly 50% with AF, practically 60% with DM, 30% with dyslipidemia, and >70% with CHF. Moreover, hypertension, DM, and dyslipidemia have been shown to not only contribute to the development of atherosclerotic cardiovascular complications but also act as crucial factors in the progression of CKD. An earlier large-scale study has also indicated that increased AF morbidity may be related to elevated serum phosphate levels (33). However, our subgroup analysis illustrated that the predictive ability of serum phosphate for mortality risk remained stable, regardless of the presence or absence of AF, CHF, DM, hypertension, and AMI. Additionally, our subgroup analysis revealed that patients without AKI had a higher mortality risk in the ICU than those with AKI (*P* for interaction = 0.033). AKI was found to be highly prevalent in critically ill patients in a previous study. Interestingly, the study also suggested that AKI is generally responsive to early intervention with acute renal replacement therapy (RRT), often leading to decreased serum phosphate levels in patients with AKI (34). Therefore, the current study patients who had an earlier AKI diagnosis might have received RRT, thereby contributing to the observed differences in our study. Consistent with a prior research finding (11), our subgroup analysis demonstrated that males had a higher all-cause mortality risk than females (*P* for interaction = 0.019). However, we also observed a notable contradictory result, wherein serum phosphate levels exhibited a significant positive correlation with ICU all-cause mortality in female patients. This contrasting result may be attributed to the unique cohort employed in our study, which was distinct from that in the previous research (11). In this study, we explicitly focused on the critically ill patient population with CHD accompanied by CKD, allowing for a detailed exploration of the link between serum phosphate and the all-cause mortality risk in this concomitant disease condition and highlighting potential gender-related differences. Nevertheless, further studies conducting comprehensive research on how serum phosphate levels correlate with the ICU and hospital death risk are warranted.

The relationship between phosphate, CHD, and CKD has garnered research attention, with a potential key mechanism being linked to atherosclerosis and medial arterial calcification. A prior study showed that a greater incidence and severity of coronary artery calcification (CAC) was associated with patients having CKD than with those not having CKD (35). Moreover, researchers have suggested that medial arterial calcification, phosphate retention, and atherosclerosis may develop concurrently with the progression of CKD (8, 36). Medial arterial calcification involves calcium phosphate deposition and osteoblastic transformation of vascular smooth muscle cells (VSMCs) (37). Initially, extracellular phosphate binds with calcium ions and fetuin-A, forming calciprotein monomers. These monomers then aggregate into primary calciprotein particles (CPPs), which develop into secondary CPPs (38, 39). The secondary CPPs further induce VSMC transformation and promote calcification (40), eventually contributing to atherosclerotic plaque instability and rupture (39–41). The inflammation-promoting function and oxidative conditions associated with CPP may accelerate atherosclerotic development, thereby escalating the risk of CVE (39, 41–43). Therefore, the increasing progression of atherosclerosis and CAC with worsening CKD is significantly correlated with the subsequent AMI and HF risk, ultimately resulting in poor prognosis (44, 45).

This study has a few limitations that should be considered. The retrospective design of our study significantly limits the ability to draw definitive causal conclusions, introducing potential selection and information biases, requiring cautious interpretation of our findings. Such a design, by its nature, can only suggest associations rather than confirm direct causal relationships. Acknowledging the complexity of clinical outcomes, we classified all-cause mortality as a secondary endpoint to reflect the nuanced impact of serum phosphate levels on patient prognosis. Moreover, potential residual confounding factors may have influenced the prognosis, even after implementing measures such as multivariate adjustment and subgroup analyses. Another limitation was that the dynamic variations in the serum phosphate levels during the hospital and ICU stays were inaccessible owing to the database limitations. Therefore, our analysis was limited to the prognosis value of baseline serum phosphate levels. Given these considerations, caution is advised when generalizing our findings to broader clinical contexts. Future studies, potentially with prospective designs and multicenter approaches, are essential to validate and extend our results.

### 2.4 Conclusions

In this study, we expanded the application of serum phosphate as an indicator in critically ill patients with CHD accompanied by CKD and showed that serum phosphate could be potentially utilized as an indicator of all-cause mortality in the ICU and hospital and during the short-term follow-up of these patients. Additionally, monitoring serum phosphate levels could help improve clinical practice decisions and health management in this patient population. However, further research is required to determine whether effective serum phosphate management can enhance the clinical prognosis of patients having CHD combined with CKD.

## Data Availability

The original contributions presented in the study are included in the article/**Supplementary Material**, further inquiries can be directed to the corresponding author.
