# Supplementary material for: Correlation between serum phosphate and all-cause mortality in critically ill patients with coronary heart disease accompanied by chronic kidney disease: a retrospective study using the MIMIC-IV database
Source: Front Cardiovasc Med. 2024 May 31;11:1371000. doi: 10.3389/fcvm.2024.1371000 (PMC11176493; doi:10.3389/fcvm.2024.1371000)
Supplement: Supplementary file 1 [file Table1.docx]

Supplementary Material

# Supplementary Tables

Supplementary Table 1 Baseline characteristics of the patients grouped into 90-day non-survivor and survivor groups.

| **Categories** | **Total** | **Non-survivors** | **Survivors** | ***P*** |
| --- | --- | --- | --- | --- |
| N | 3557 | 945 | 2612 |  |
| Gender |  |  |  |  |
| Female | 1152 (32.4%) | 302 (32%) | 850 (32.5%) | .773 |
| Male | 2405 (67.6%) | 643 (68%) | 1762 (67.5%) |  |
| Age, years | 77.1 (69.0–84.2) | 81.1 (73.1–87.5) | 75.7 (67.5–82.9) | <.001 |
| Height, cm | 170.0 (160.0–178.0) | 168.0 (157.0–175.0) | 170.0 (160.0–178.0) | <.001 |
| Weight, kg | 80.6 (68.5–95.2) | 75.5 (64.2–89.5) | 82.7 (70.0–97.0) | <.001 |
| BMI, kg/m^2^ | 28.3 (25.1–32.3) | 27.1 (23.9–30.9) | 28.8 (25.6–32.8) | <.001 |
| SBP, mmHg | 115.7 (106.5–127.5) | 111.4 (103.4–122.8) | 117.1 (108.0–129.6) | <.001 |
| DBP, mmHg | 57.8 (51.6–65.0) | 57.9 (51.0–64.4) | 57.6 (51.9–65.2) | .205 |
| MBP, mmHg | 74.2 (68.6–80.6) | 72.7 (67.2–79.1) | 74.6 (69.0–81.0) | <.001 |
| Heart rate, bpm | 79.2 (70.4–88.8) | 82.6 (70.9–93.1) | 78.4 (70.2–87.4) | <.001 |
| Comorbidities, n (%) |  |  |  |  |
| AKI | 947 (26.6%) | 338 (35.8%) | 609 (23.3%) | <.001 |
| AF | 1633 (45.9%) | 495 (52.4%) | 1138 (43.6%) | <.001 |
| Chronic pulmonary disease | 1121 (31.5%) | 323 (34.2%) | 798 (30.6%) | .044 |
| Cerebrovascular disease | 565 (15.9%) | 184 (19.5%) | 381 (14.6%) | <.001 |
| CHF | 2303 (64.7%) | 682 (72.2%) | 1621 (62.1%) | <.001 |
| DM | 1981 (55.7%) | 475 (50.3%) | 1506 (57.7%) | <.001 |
| Dyslipidemia | 1064 (29.9%) | 259 (27.4%) | 805 (30.8%) | .055 |
| Liver disease | 295 (8.3%) | 123 (13%) | 172 (6.6%) | <.001 |
| Hypertension | 3278 (92.2%) | 856 (90.6%) | 2422 (92.7%) | .042 |
| Mechanical ventilation | 1268 (35.6%) | 366 (38.7%) | 902 (34.5%) | .023 |
| MI | 1854 (52.1%) | 520 (55%) | 1334 (51.1%) | .041 |
| Peripheral vascular disease | 866 (24.3%) | 256 (27.1%) | 610 (23.4%) | .025 |
| Respiratory failure | 1010 (28.4%) | 451 (47.7%) | 559 (21.4%) | <.001 |
| Rheumatic disease | 144 (4%) | 39 (4.1%) | 105 (4%) | .963 |
| Laboratory tests |  |  |  |  |
| RBC, #/uL | 3.4 (3.0–3.8) | 3.3 (2.9–3.8) | 3.4 (3.0–3.8) | .005 |
| WBC, K/uL | 10.8 (8.2–14.6) | 11.6 (8.4–16.2) | 10.6 (8.1–14.0) | <.001 |
| Platelets, K/uL | 178.5 (135.5–237.5) | 185.0 (131.0–251.5) | 177.0 (136.0–233.2) | .228 |
| Hematocrit, % | 30.1 (27.0–34.4) | 30.6 (26.9–34.6) | 30.0 (27.1–34.4) | .438 |
| Hemoglobin, g/dL | 9.8 (8.7–11.2) | 9.8 (8.6–11.2) | 9.8 (8.8–11.2) | .062 |
| eGFR, ml/min/1.73m^2^ | 48.1 (30.8–65.5) | 42.0 (26.2–61.3) | 50.2 (33.0–67.3) | <.001 |
| Serum creatinine, mg/dL | 1.4 (1.1–2.0) | 1.5 (1.1–2.3) | 1.3 (1.1–1.9) | <.001 |
| Anion gap, mmol/L | 15.5 (13.0–18.0) | 17.0 (15.0–20.0) | 15.0 (13.0–17.5) | <.001 |
| Serum bicarbonate, mEq/L | 22.5 (20.0–25.0) | 21.5 (19.0–24.5) | 22.5 (20.5–25.0) | <.001 |
| BUN, mg/dL | 37.0 (25.5–56.0) | 47.0 (32.0–67.5) | 33.5 (24.0–51.5) | <.001 |
| Urine Output, ml/24h | 1320.0 (727.0–2115.0) | 963.0 (400.0–1565.0) | 1450.0 (875.0–2261.0) | <.001 |
| Serum calcium, mg/dL | 8.4 (8.1–8.9) | 8.4 (8.0–8.9) | 8.4 (8.1–8.9) | .403 |
| Serum chloride, mEq/L | 103.5 (99.0–107.0) | 102.0 (97.5–106.0) | 104.0 (100.0–107.5) | <.001 |
| Glucose, mg/dL | 136.5 (111.5–178.0) | 147.5 (117.0–192.0) | 133.0 (110.5–171.8) | <.001 |
| Serum sodium, mEq/L | 138.5 (135.5–141.0) | 138.0 (135.0–141.0) | 138.5 (136.0–140.5) | .125 |
| Serum potassium, mEq/L | 4.4 (4.0–4.9) | 4.4 (4.0–5.0) | 4.4 (4.0–4.8) | .033 |
| INR | 1.3 (1.1–1.5) | 1.4 (1.2–1.8) | 1.2 (1.1–1.4) | <.001 |
| PT | 14.2 (12.6–16.6) | 15.1 (13.1–19.4) | 13.9 (12.6–15.9) | <.001 |
| PTT | 33.5 (28.6–47.0) | 36.8 (29.6–55.0) | 32.7 (28.3–44.5) | <.001 |
| Serum phosphate, mg/dL | 3.9 (3.3–4.7) | 4.2 (3.5–5.2) | 3.8 (3.3–4.5) | <.001 |
| APS III | 49.0 (40.0–61.0) | 58.0 (48.0–74.0) | 46.0 (38.0–57.0) | <.001 |
| SOFA | 6.0 (4.0–8.0) | 7.0 (5.0–10.0) | 5.0 (3.0–7.0) | <.001 |
| LODS | 5.0 (4.0–7.0) | 7.0 (5.0–9.0) | 5.0 (3.0–7.0) | <.001 |
| OASIS | 32.0 (26.0–38.0) | 36.0 (29.0–42.0) | 30.0 (25.0–36.0) | <.001 |
| SAPS II | 41.0 (35.0–50.0) | 48.0 (40.0–58.0) | 39.0 (33.0–47.0) | <.001 |

Abbreviation: BMI, body mass index; SBP, systolic blood pressure; DBP, diastolic blood pressure; MBP, mean blood pressure; AKI, acute kidney injury; AF, atrial fibrillation; CHF, congestive heart failure; DM, diabetes mellitus; MI, myocardial infarction; eGFR, estimated glomerular ﬁltration rate; RBC, red blood cell; WBC, white blood cell; BUN, blood urea nitrogen; INR, international normalized ratio; PT, prothrombin time; APTT, activated partial thromboplastin time; APS III, acute physiology score III; SOFA, sequential organ failure assessment; LODS; OASIS, oxford acute severity of illness score; SAPS II, Simplified acute physiological score II.
